# A qualitative study of pain and related symptoms experienced by people with Ehlers-Danlos syndromes

**DOI:** 10.3389/fmed.2023.1291189

**Published:** 2024-01-03

**Authors:** Jane R. Schubart, Susan E. Mills, Clair A. Francomano, Heather Stuckey-Peyrot

**Affiliations:** ^1^Department of Surgery, Penn State College of Medicine, Hershey, PA, United States; ^2^Department of Medical and Molecular Genetics, Indiana University School of Medicine, Indianapolis, IN, United States; ^3^Department of Medicine, Penn State College of Medicine, Hershey, PA, United States

**Keywords:** Ehlers-Danlos syndromes, hereditary disorders of connective tissue, aging, self-care, symptoms, longitudinal, qualitative

## Abstract

**Introduction:**

Individuals with Ehlers-Danlos syndromes (EDS) often have complex and multi-faceted symptoms across the lifespan. Pain and the related symptoms of fatigue and sleep disorders are common. The objective of this qualitative study was to understand how participants manage their pain and related symptoms.

**Methods:**

The design was a qualitative thematic content analysis. Twenty-eight interviews were conducted to collect data from individuals who were participants in a prior quantitative longitudinal study. A semi-structured interview guide was designed to focus on and understand the trajectory of pain, sleep, fatigue, and general function. The interview continued with questions about coping mechanisms and obstacles to maintaining a sense of well-being.

**Results:**

Symptoms reported by participants were widespread and often interwoven. Pain was universal and often resulted in fatigue and disordered sleep which impacted physical function. Most participants reported that their symptoms worsened over time. Participants reported a wide range of effective interventions and most reported developing self-care strategies to adapt to their disabilities/limitations. Solutions included complementary interventions discovered when conventional medicine was unsuccessful. Very few relied on a “system” of health care and instead developed their own strategies to adapt to their disabilities/limitations.

**Discussion:**

EDS symptoms are often debilitating, and their progression is unknown. For most participants, symptoms worsened over the time. Even though participants in our study, by experience, were self-reliant, the importance of knowledgeable medical providers to help guide self-care should be emphasized.

## 1 Introduction

The Ehlers-Danlos syndromes (EDS) are hereditary disorders of connective tissue generally characterized by joint laxity and skin involvement (1, 2). Because every organ system may be involved, patients experience a myriad of clinical symptoms, including autoimmune and autonomic nervous system disorders, headaches, gastrointestinal and bladder dysfunction, and peripheral neuropathy. Chronic pain and related symptoms of fatigue and sleep disorders are common (3–7). Patients with EDS are often predisposed to trauma including subluxations, dislocations, chronic arthralgia, and soft-tissue inflammation that often result in disabling musculoskeletal disorders.

The natural history of the most common type of EDS, known as hypermobile EDS, has been described as progressing over three overlapping phases. Ligament laxity and frequent dislocations and subluxations often begin in childhood, with or without complaints of pain. In young adults, pain worsens. Later in life, individuals often experience stiffness, restricted joint motion and chronic pain (8). Unfortunately, there is no known cure and no standardized treatment. Symptoms management may include a combination of treatments including conventional medicine, physical therapy, exercise, lifestyle changes, and as last resort, surgery. Chronic pain is one of the most common complaints for patients with EDS (6, 9). Pain can manifest as musculoskeletal, visceral, and/or neuropathic. The mechanisms include repeated soft tissue damage from frequent dislocations, recurrent micro-traumas at the joint surface, compression of the spinal cord from cervical spine laxity, and fragility of vasculature and visceral organs (10). Widespread pain in EDS has been associated with central sensitization (11, 12). Fatigue, depression, and anxiety may intensify pain for EDS patients (5, 9, 10). The hormonal environment may also play a role in the perception of pain and response to analgesics, however prospective studies are needed in the EDS population (13). Given the complexity of EDS, patients often need treatment for their pain and related symptoms, which may be further complicated by the aging process.

In a prior qualitative study, Bennett et al. (14) conducted a thematic synthesis of the published literature using eight online databases (1990–2018) and reported on nine studies of participants’ lived experiences. The main themes reported were lack of medical professional understanding; restricted life; social stigma; “trying to keep up”; and gaining control (14). Baeza-Velasco et al. (15) conducted a narrative review of studies focusing on psychological factors associated with pain chronicity and disability. The authors concluded that chronic pain conditions in EDS should be better understood before integrating EDS patients into chronic pain management programs (15). Anderson and Lane (16) synthesized 13 qualitative studies to understand the reasons for diagnostic delay and reported four thematic categories: disease, patient, provider, and system. Berglund et al. (17) interviewed 11 participants from a Swedish support group. Grounded theory analysis revealed a main theme of “living a restricted life.” Schmidt (18) used semi-structured interviews and interpretative phenomenological analysis to explore the decisions of 11 women attending a pain clinic in the United Kingdom. Themes included the unpredictability of pain and keeping pain to a manageable level by considering whether the planned activity was worth it (18). Simmonds et al. (19) in a mixed methods study recruited 946 participants from the Hypermobility Syndromes Association and EDS Support United Kingdom to investigate exercise beliefs and behaviors. Key themes emerged regarding the role of physiotherapy, including hands-on guidance and feedback (19). Palomo-Toucedo et al. (20) conducted interviews with 25 individuals with EDS, recruited from patient groups in Spain. This study focused on psychosocial impact (20). Doyle and Halverson (21) interviewed 24 patients from one US clinic and focused on complementary and alternative medicine and explored changing attitudes toward pain in 15 patients (22). Bennett et al. (23) conducted interviews with nine women in the United Kingdom to investigate self-management strategies.

This qualitative study builds on our prior quantitative study (24) where we report over a decade of longitudinal symptoms data in the EDS population. In the prior study, we extended a cross-sectional data collected and assembled between 2001 and 2013 under a protocol entitled *Clinical and Molecular Manifestations of Heritable Disorders of Connective Tissue* by the National Institute on Aging (NIA) Intramural Research Program, National Institutes of Health (25). Our research goal was to examine symptoms changes over time in participants with a diagnosis of EDS for whom baseline data is available and who had a comprehensive physical examination at their baseline NIA visit. We examined changes from their baseline NIA clinic visit to follow-up survey in pain, sleep, fatigue, and overall function. Our study cohort included 91 participants who had completed at least one of the following surveys: Brief Pain Inventory (BPI) (26), Pittsburgh Sleep Quality Index (PSQI) (27), Multidimensional Fatigue Inventory (MFI-20) (28), and Short Form (SF-36) Health Survey (29), at baseline and follow-up. We used mixed effects linear regression models to examine the change in scores for multiple indices reported by participants. The most important observation was wide heterogeneity between reported individual experiences. The objective of the present study is to expand on our previous research and to understand what factors contributed to the participants’ symptoms changes over time and how they adapted accordingly.

## 2 Materials and methods

### 2.1 Design and sampling method

A qualitative study was conducted to collect data from individuals who are participants in a longitudinal study. The design was a thematic content analysis. We identified participants from the prior study who had completed the outcome measures for pain, sleep, fatigue, and function. This sampling strategy stemmed logically from the research questions being addressed and provided the possibility to draw creditable explanations from the data. We selected the first 30 participants who were contactable (i.e., current phone number). We contacted them by phone and then sent an email describing the study. Informed consent was obtained prior to data collection. All participants agreed to video-conferencing.

### 2.2 Data collection

We used individual interviews to answer the research question, “What factors contributed to patient self-reported changes in their health status over time?” All interviews were conducted by one experienced interviewer. A semi-structured interview guide was used. Interviews began with questions about general function, pain, sleep, fatigue, and the trajectory EDS symptoms, and continued with questions about coping mechanisms and obstacles to maintaining a sense of well-being. (Supplementary Appendix A) Field notes were taken. Interviews lasted between 45 and 60 min and were audio-recorded. Participants were not compensated.

### 2.3 Data analysis

Interview audio recordings were professionally transcribed verbatim. Data were analyzed using qualitative thematic content analysis. The codebook development process included the following steps: (1) three researchers independently coding three transcripts each; (2) two researchers independently coding 28% of the interviews to achieve the desired 0.79 kappa statistic; (3) one researcher coding the remaining transcripts. We used MAXQDA (version 22.3.0) software to examine relationships between codes/categories to determine initial themes using both an *a priori approach* and an *inductive approach* (30). Our interview guide provided the initial framework. We used “open coding,” an inductive approach to discover themes that we did not anticipate before analyzing the data. Theme discovery included searching for recurring concepts and topics, analogies, and metaphors that the participants used to represent their experiences (31). During this iterative process some themes were combined, and some were found irrelevant even though they seemed very interesting. Three authors (Mills, Schubart, Stuckey-Peyrot) were immersed in the data and participated in this process. The fourth author (Francomano) reviewed and affirmed the overarching themes.

### 2.4 Rigor and reproducibility

Methods to ensure the study’s rigor included strategies to address credibility, dependability, confirmability, and transferability (32). Credibility was heightened by using a semi-structured interview guide developed with input from people with EDS. All authors have expertise in qualitative research and/or the clinical care of patients with EDS. Dependability was accomplished by adhering to established qualitative research methods. We narrowed our focus to specific lines of inquiry. Confirmability was achieved through a sample of participants who provided feedback on the findings. We adhered to a carefully constructed codebook, achieved excellent interrater reliability, data saturation, and found consistency in the interviews. Because the study was a qualitative design, we do not know the frequency of the viewpoints heard and themes derived in the larger EDS population, however, transferability was enhanced by providing a description of the study participants’ characteristics and lived experiences. Hence, the results are likely applicable to people with EDS and other heritable disorders of connective tissue.

## 3 Results

### 3.1 Sample description

Thirty individuals agreed to participate initially, however, one was unable to follow through due to physical health decline. Thus, 29 interviews were conducted (3 male, 26 female). All participants were white. One recording was not analyzable for technical reasons. Thus, 28 were transcribed and analyzed. The characteristics of the study sample are shown in Table 1.

**Table 1:** Characteristics of the study sample.

Participant #	Age (years)^*^	Sex^†^	EDS diagnosis^‡^	Date of NIA study visit^§^	State^†^
30	33	F	Classical	2004	OH
31	47	F	Classical	2004	ONT/CANADA
44	67	F	Hypermobile	2005	WI
65	68	F	Classical	2004	OH
76	69	F	Classical	2004	MD
79	52	F	Hypermobile	2004	MD
80	36	F	Hypermobile	2004	MD
81	64	M	Hypermobile	2004	MD
82	30	F	Hypermobile	2004	MD
83	33	M	Hypermobile	2004	MD
109	71	F	EDS, not specified	2006	CA
116	58	F	Vascular	2007	FL
117	85	M	EDS, not specified	2007	FL
118	58	F	Classical	2007	AZ
121	56	F	EDS, not specified	2007	MI
130	67	M	EDS, not specified	2007	PA
145	72	F	Hypermobile	2006	RI
176	66	F	EDS, not specified	2007	FL
223	76	F	Hypermobile	2008	IN
228	74	F	EDS, not specified	2008	OH
240	63	F	EDS, not specified	2008	CA
245	48	F	Vascular	2008	TX
292	74	F	EDS, not specified	2011	PA
824	64	F	EDS, not specified	2008	MD
1130	29	F	EDS, not specified	2009	PA
1204	42	F	EDS, not specified	2011	NC
1241	32	F	EDS, not specified	2012	MD
1249	60	F	Hypermobile	2012	MD

^*^At the time of the study interview.

^†^As reported in the original NIA study (predates the current 2017 diagnostic criteria).

^‡^All participants were diagnosed by the principal investigator of the original NIA study (categories included hypermobile, classical, vascular, not specified).

^§^Year of entry to NIA study.

### 3.2 Characteristics of the cohort: symptoms of EDS

The symptoms reported by participants were widespread. Pain, fatigue, and sleep disruption impacted general function. Because individuals with EDS often experience spontaneous dislocations/subluxations, pain is often related to joints. One participant said, “I have dislocated everything that could be dislocated. Even my larynx is dislocated.” (#824) Another said, “I live every day of my life in pain.” (#176) Participants experienced many overlapping conditions.

“The biggest problem tends to be hip dislocations for me. I’ll have a hip dislocation. And I then I’m not quite an invalid, but I’m extremely mobility limited and in a lot of pain. And significantly reduced capacity for anywhere from a week to a month.” (#83).

“… my joints are totally worn out. How much of that is the EDS? How much is the psoriatic arthritis? How much is the osteoarthritis? Now, I just do not know.” (#76).

“My joint pain increased so much in the last two years … my stamina is much less. I’m functional, but I’m not comfortably functional.” (#79).

### 3.3 Introduction to themes

Most participants reported that their general function declined over time. However, to the extent possible, most identified ways of coping with their physical challenges without relying on the health care system. Reported solutions included alternative strategies when conventional medicine did not meet their needs. These decisions were often dependent on ability to pay for treatments. The result was a process of care that was patient-driven and a testament to the strength of their resilience. We did not find differences among the different EDS diagnosis types.

#### 3.3.1 Theme 1: persistence of pain and other symptoms

Although there was variability in participants’ symptoms over time, most were physically declining and continued to deal with pain and other symptoms. It is difficult for patients to know whether a decline is due to EDS, normal aging, or both. One participant (age 58) noted, “Most of the people I know, who are my age, do not feel the way I do.” (#116) Although most reported a decline in their health status, some described fluctuations between severe and mild symptoms. Others experienced little respite from the cycle or described a “downward phase” characterized by uncontrolled pain and increasing acute episodes requiring urgent treatment. One participant said, “I’m so frustrated because [INHALE] I’m exhausted. And I’m tired. And I always feel like there’s something more going on.” (#121).

“The last five years it’s really declined a lot. And then this past year, it’s agonizing as well as declining. And I have a lot of deep fatigue. Horrible insomnia, loads of problems with my muscles, my tendons, my nerves.” (#117).

“I feel it’s like a roller coaster. Like, I get worse for some months. And then function increases for a while, and then you dip sometimes lower. And then sometimes a little bit higher and then lower that way.”

“I would say it comes and goes. I do not even want to say it’s a day-to-day thing. It’s more of a moment-to-moment thing. There’re times when my hip will be killing me, and for like half an hour. And then an hour later, I’m like, oh, I feel fine now.” (#30).

“I do not realistically see my condition improving over time … it seems predictable that it will continue to decline over time, which is unfortunate, but seems likely.” (#1130).

#### 3.3.2 Theme 2: lifestyle modifications and integrative approaches

Participants reported a wide range of effectiveness of interventions. Alternative therapies appear to have been more effective than prescribed medications and elective surgery. Physical therapy was often beneficial. Complementary therapies were frequently mentioned as beneficial (e.g., acupuncture, chiropractic, meditation, mindfulness, medical marijuana).

“I just kind of feel miserable every day. In fact, I’m sitting here today with a pain patch on my neck. It’s just Chinese herbs.” (#79).

Participants reported multiple surgeries (i.e., #145 had 26 surgeries, #76 had 24, #1241 had 20), predominantly neurosurgery and orthopedic surgeries, but none provided evidence that the surgeries were successful. For example, one participant had a complicated ankle repair that needed several subsequent surgeries (#81).

Very few relied on a “system” of health care and instead developed their own strategies to adapt to their disabilities/limitations. Strategies included exercise and diet modification, and solutions to help sleep quality (i.e., sleep positions, pillows, and pacing activities). Strategies reported to manage symptoms also included trial and error with assistive devices such as braces, cervical collars, finger splints, casts, dental appliances, TENS, and sleep aid devices. Some of these strategies may be too expensive for people who do not have insurance.

#### 3.3.3 Theme 3: fragmented care

Surprisingly, most participants did not view a physician as their central source of care, and they did not have access to an integrated system of care. In most cases, care was fragmented. For example, EDS frequently involves all joints and patients often need multiple orthopedic specialists. Without an integrated system, patients need to navigate a complex health care landscape. Participants found individual providers to treat their symptoms (e.g., physical therapist, neurologist, rheumatologist, etc.).

“When it [neck] was super-duper bad, and I could not move, and it was scary, and I probably should have seen somebody, but I only trust him [specialist].” (#79).

To the extent possible, they tended to identify solutions to their physical challenges, rather than relying on the health care “system.”

“I make a lot of coconut oil cannabis, and make up a little gummy bears … helping with sleep and pain and, stuff like that.” (#292).

“It’s hard to find a position for work where I am not in pain or uncomfortable in some way. I think I would have to be almost reclined and supported to have zero pain. But I’m working too much at the computer. So, to get up and move around does seem to help a little bit.” (#118).

“I do have a primary care doctor … I see them about yearly for physicals sort of thing. So, I generally am not going to my primary care doctor expecting them to provide useful answers for me … I would say that I generally have not cracked the problem of how to receive effective health care as an EDS patient.” (#83).

#### 3.3.4 Theme 4: coping

Participants generally demonstrated the ability to cope with traumatic and stressful events. Most participants described specific ways to self-manage some aspects of their care. They found ways to overcome the obstacles created by EDS, adapt to their changing health needs and identify coping strategies. They reported various home accommodations, such as stair lift (#76), moving to a one-story house (#116), and balancing household tasks to conserve their energy. (#130).

“I have, like, 42 different chairs. Switch out chairs all the time. Stand up, sit down, move to the bed. I have a big desk that moves on my bed. I have shoes of every variety.” (#80).

Support systems and social networks were important. Some were able to negotiate workplace accommodations. Others mentioned reliance on family support (#44, #83), friends (#80) and support groups, and one mentioned social media (#44). Several mentioned “pushing through” their symptoms, even though this strategy may have a negative impact. For example, one participant said, “I’m used to pushing through it … but it can exacerbate symptoms sometimes. (#292).

“At this point, kind of feel like I’m beholden to my pain, even though I try to push through it and plan social activities and keep those social activities. I find I often have to cancel on people because I just cannot quite manage to summon up the energy because the pain took it all, so to speak.” (#1130).

Participants tried to make better choices and decisions. One summed this up as “constantly shifting priorities back to what supports you.” (#118). This process of adaption to changing health that came with maturity resulted in improved quality of life.

“I think there was a period of improvement between the initial study contact and, I would say about, 12 to 14 years ago. … I’ve sort of made a little bit more peace with the things that I can and cannot do. A little of that may be maturity with age. I’ve stopped doing some dumb things.” (#31).

“I love, at the age of 71 -- I’ll be 72 in April. I’ve actually improved in my quality of my life.” (#145).

## 4 Discussion

Our sample represents a cohort of longitudinal study participants who were diagnosed with EDS and eligible for the original NIA study. Most reported that their general function declined over time due to their chronic pain. Although most did not rely on the formal healthcare system, they were able to identify various solutions to their physical challenges.

Unlike reports in other qualitative studies focusing on EDS (26, 28, 29), our participants did not mention that their condition was poorly understood or dismissed by health care professionals. These participants have been involved with EDS research for several decades and are likely better connected to knowledgeable medical practitioners than most. The results of our study indicated a patient-driven process that was not constrained by expectations that the health care system was going to take care of their needs. Participants cited multiple reasons for seeking care outside the conventional health care system, including their ability (or inability) to pay for clinical care and difficulty accessing appropriate specialists. Many accessed providers of specialized care only when unavoidable (e.g., orthopedic interventions, ruptured internal organ repair). They expressed a pragmatic view of the health care system. Their solutions included interventions such as mindfulness and physical therapy to alleviate pain.

Our study suggests that people with EDS experience pain cycles of decline and reprieve, and that these cycles are often influenced by periods of time when self-care is not as rigorous. Metaphors are often used by patients to describe pain (33). Our study participants most often expressed their chronic pain in terms such as cycles, seasons, and roller coaster rides. Reliance on self-care without accessing the health care system may have detrimental consequences. For example, when patients do not feel connected to a system of medical care, they may not get necessary screening (e.g., optimal pain management and bone density scan to detect early osteoarthritis).

Our main findings were that individuals living with EDS demonstrated a high degree of self-reliance and low expectations that the health care system would solve their problems. This contrasts with findings in other studies. For example, Berglund et al. (17) revealed a main theme of “living a restricted life” that may explain ways in which “fears, pain, stigmatization, and experiences of “non-affirmation” in health care may impact the quality of daily living and social life. Unlike findings in some prior qualitative studies (21), our participants generally continued to work despite significant disability, and had adapted and negotiated their work environment and the health care system’s formidable barriers. Consistent with the literature, our study identified a wide range of individual differences in coping with chronic illness effects (32), and the use of integrative medicine strategies (23).

Resilience has been previously described as a path to adaptation to stressors such as chronic illness (34–36). Faced with threatening life events such as a serious diagnosis, individuals adapt to a new reality (37). There is no standardized treatment and no known cure for EDS. Given the dearth of longitudinal clinical data, there is no predictable trajectory. Recent studies have acknowledged the positive influence of resilience on adjustment to chronic pain (35). This is particularly relevant because our data suggests that pain is a principal manifestation of EDS across the lifespan.

### 4.1 Limitations

Our study included a longitudinal cohort who had been living with an EDS diagnosis for at least a decade. A limitation was that the cohort was exclusively white and predominately female, a feature consistent with EDS research studies at large. The participants’ insurance and financial status were not known. There is potential selection bias. We were not able to contact some eligible individuals (assumed address and/or emails changed). There were no refusals, one person was too sick to participate, and one did not have internet connectivity. While the longitudinal design is a strength, a limitation is that there may have been recall bias because over a decade had passed since participants first entered the study. The sampling strategy was purposeful because all participants were enrolled in the prior NIA study and were “experienced” patients, however, this convenience sampling strategy has limitations. We do not know the impact of type of EDS or age, or whether the conclusions are generalizable to people living with EDS more broadly.

### 4.2 Implications for clinical practice and health policy

This study demonstrates the importance of (1) self-care and (2) the ability to navigate the complexity of the healthcare system. The participants in this study reported their reliance on self-care versus accessing appropriate clinical care. Most patients with EDS experience generalized joint hypermobility, chronic widespread pain, and fatigue (38). Pain treatment is complex and may require coordinated care that includes intensive physical therapy, occupational therapy, and bracing.

There are not enough providers trained to recognize and take care of these complex patients. Clinical practice guidelines are lacking and there is no consensus on the best practice for medical surveillance and medical and surgical interventions. The health care system frequently imposes additional barriers, including limited clinic visit time and complex billing structures. For example, patients with EDS may be referred to specialty care providers whose expensive services are not covered by their insurance plan. Many cannot afford these services and therefore must rely on their own resourcefulness to manage their symptoms. This study has highlighted the importance for balance between patient resourcefulness and competent providers to manage their overall clinical care.

## 5 Conclusion

For most participants in our study, symptoms worsened over the time. They were often self-reliant, but many lacked access to appropriate healthcare to manage their clinical needs. Recognizing the complexity of EDS, self-care should not be a substitute for utilizing knowledgeable healthcare professionals.

## Data availability statement

The datasets presented in this article are not readily available because this is a qualitative study. Aggregate data can be made available upon reasonable request to the corresponding author. Ethics approval prohibits sharing of individual-level data. Requests to access the datasets should be directed to jschubart@pennstatehealth.psu.edu.

## Ethics statement

This study involving human subjects was approved by the Human Subjects Research Protection Program (HRPP), the Institutional Review Board for Penn State College of Medicine. The study was conducted in accordance with local legislation and institutional requirements. The participants provided their verbal consent to participate in the study which was confirmed and documented.

## Author contributions

JS: Conceptualization, Funding acquisition, Methodology, Project administration, Writing – original draft, Writing – review & editing. SM: Conceptualization, Data curation, Formal analysis, Investigation, Software, Writing – original draft, Writing – review & editing. CF: Validation, Writing – review & editing. HS-P: Formal analysis, Methodology, Resources, Software, Validation, Writing – original draft, Writing – review & editing.
