# Supplementary material for: A qualitative study of pain and related symptoms experienced by people with Ehlers-Danlos syndromes
Source: Front Med (Lausanne). 2024 Jan 3;10:1291189. doi: 10.3389/fmed.2023.1291189 (PMC10792024; doi:10.3389/fmed.2023.1291189)
Supplement: Supplementary file 1 [file Data_Sheet_1.docx]

**SUPPLEMENTAL MATERIAL**

**APPENDIX A**

INTERVIEW GUIDE

The focus of this interview is on general function, pain, sleep, and fatigue, and the changes over time.

Let’s start with general function. I’m going to show a commonly used measure of daily function and the ability to perform routine tasks.

*Show Karnofsky Performance Scale.* Please pick the category that best fits your function today.

**Now let’s talk about pain.**

Compared to when you completed the previous survey questionnaire __ years ago, do you feel that your pain has improved, worsened or stayed about the same?

Pick the 3 areas of your body where you have the most pain.

Tell me about the type of pain you have in *location 1, …*

Prompts: Frequency, how often? Is it constant, or does you pain come and go? Severity?

How has your pain interfered with: Sleep? Ability to work? Ability to participate in social activities?

What have you been doing about pain? (Prompts: medications (ask follow-up questions), surgery, PT, acupuncture, mindfulness, lifestyle)

What has worked, what hasn’t?

**Let’s talk about sleep.**

Describe the quality of your sleep now. PROBE: Do you sleep through the night? If you wake, what are the reasons? Using any strategies to help?

**Let’s talk about fatigue.**

How is your stamina (and/or fatigue) now? Using any strategies to help?

**What has been the trajectory of your EDS symptoms?**

Have you felt improvement over time?

Or have you felt that there has been no change?

Or have you noticed a decline since young adult?

**What have been your coping mechanisms?**

PROBE: adapting to changing health, social support, resilience

**What are other things you do to maintain a sense of well-being?**

PROMPT: Work, hobbies, volunteering, social networking, exercise, other?

**What are the main obstacles to maintaining your sense of well-being?**

PROMPTS: Other medical, economic or social issues?
